# Laser‐driven radiation: Biomarkers for molecular imaging of high dose‐rate effects

**DOI:** 10.1002/mp.13741

**Published:** 2019-08-29

**Authors:** Theodor Asavei, Mariana Bobeica, Viorel Nastasa, Gina Manda, Florin Naftanaila, Ovidiu Bratu, Dan Mischianu, Mihail O. Cernaianu, Petru Ghenuche, Diana Savu, Dan Stutman, Kazuo A. Tanaka, Mihai Radu, Domenico Doria, Paul R. Vasos

**Affiliations:** ^1^ Extreme Light Infrastructure ‐ Nuclear Physics ELI‐NP “Horia Hulubei” National Institute for Physics and Nuclear Engineering 30 Reactorului Street RO‐077125 Bucharest‐Magurele Romania; ^2^ National Institute for Laser, Plasma and Radiation Physics 409 Atomistilor Street RO‐077125 Bucharest‐Magurele Romania; ^3^ Cellular and Molecular Medicine Department “Victor Babes” National Institute of Pathology 99‐101 Splaiul Independentei Bucharest 050096 Romania; ^4^ Carol Davila University of Medicine and Pharmacy Bucharest Dr Carol Davila Central Mil University Emergency Hospital 88th Mircea Vulcanescu Str Bucharest Romania; ^5^ Amethyst Radiotherapy Clinic Dr Odaii 42 Otopeni Romania; ^6^ Department of Life and Environmental Physics Horia Hulubei” National Institute for Physics and Nuclear Engineering 30 Reactorului Street RO‐077125 Bucharest‐Magurele Romania; ^7^ Johns Hopkins University 3400 N Charles St Baltimore Maryland 21218 USA; ^8^ Centre for Plasma Physics, School of Mathematics and Physics Queen's University Belfast Belfast BT7 1NN United Kingdom; ^9^ Research Institute of the University of Bucharest (ICUB) 36‐46 B‐dul M. Kogalniceanu RO‐050107 Bucharest Romania

**Keywords:** biomarkers, free radicals, high dose‐rate radiation, laser‐driven particles, molecular imaging, radiobiology, reactive molecular species

## Abstract

Recently developed short‐pulsed laser sources garner high dose‐rate beams such as energetic ions and electrons, x rays, and gamma rays. The biological effects of laser‐generated ion beams observed in recent studies are different from those triggered by radiation generated using classical accelerators or sources, and this difference can be used to develop new strategies for cancer radiotherapy. High‐power lasers can now deliver particles in doses of up to several Gy within nanoseconds. The fast interaction of laser‐generated particles with cells alters cell viability via distinct molecular pathways compared to traditional, prolonged radiation exposure. The emerging consensus of recent literature is that the differences are due to the timescales on which reactive molecules are generated and persist, in various forms. Suitable molecular markers have to be adopted to monitor radiation effects, addressing relevant endogenous molecules that are accessible for investigation by noninvasive procedures and enable translation to clinical imaging. High sensitivity has to be attained for imaging molecular biomarkers in cells and *in vivo* to follow radiation‐induced functional changes. Signal‐enhanced MRI biomarkers enriched with stable magnetic nuclear isotopes can be used to monitor radiation effects, as demonstrated recently by the use of dynamic nuclear polarization (DNP) for biomolecular observations *in vivo*. In this context, nanoparticles can also be used as radiation enhancers or biomarker carriers. The radiobiology‐relevant features of high dose‐rate secondary radiation generated using high‐power lasers and the importance of noninvasive biomarkers for real‐time monitoring the biological effects of radiation early on during radiation pulse sequences are discussed.

## 1 Introduction

It is currently estimated that more than 50% of all cancer patients can benefit from radiotherapy at some stage during their treatment in order to maximize disease control and survival.^1^, ^2^, ^3^ Specifically, throughout the world, every year, radiotherapy could increase survival for approximately 3.5 million cancer patients and another 3.5 million patients could undergo palliative care based on radiation treatment.^4^ The efficacy of radiation‐based treatment in cancer is highly dependent on the type and extent of damage induced by ionizing radiation in tumor cells, as well as on their ability to repair radiation‐induced disruptions. Cells are damaged by radiation via: (a) the direct interaction between ionizing radiation and cellular components such as DNA, and (b) the generation of highly reactive species that propagate damage inside and outside the irradiated area (via bystander effects). Both mechanisms alter homeostatic molecular pathways and, consequently, cell viability.^5^ The generation of reactive oxygen and nitrogen molecular species (ROS, RNS) is a critical component of radiation antitumor cell effects. Oxidative and nitrosative bursts can reshape irreversibly cellular components such as nucleic acids, membranes, and proteins, and affect the redox state within cells by altering the ratio of oxidizing to reducing equivalents. The main pathways affected by the presence of ROS are related to redox signaling.^6^ These electron‐transfer mediated cellular processes are activated by molecular dioxygen‐originating ROS formed via different reactions, such as superoxide anion, hydroxyl radical, or hydrogen peroxide.^6^ It is noteworthy that the same ROS that are cytotoxic via redox‐sensitive moieties such as thiol groups, which may alter protein structures at high concentrations^7^ sustain cellular homeostasis at lower concentrations. The process is controlled by complex antioxidant mechanisms.^8^, ^9^ Cells are endowed with protection mechanisms against radiation‐induced damage such as antioxidant mechanisms, DNA damage repair, cell cycle arrest, and unfolded protein response. These cellular mechanisms act to protect healthy tissue, but the same processes can have a negative impact on the efficacy of cancer radiotherapy.

Using rapidly developing omics approaches, the various pathways involved in the cells’ coping with radiation‐induced damage have been studied and molecular endpoints for these pathways are often analyzed (e.g., BCL2, p53, TGF‐β, NF‐κβ, c‐MET, PI3K/Akt). The evaluation of cellular responses to the radiation challenge relies on the detection of a broad array of accumulating products of cellular pathways activated by irradiation. The final outcome of this network of events can be defined *in vitro* and *ex vivo* through omics tools, and molecular biomarkers can be validated by microscopy, spectroscopy, and chromatography. Despite the progress in revealing the mechanisms underlying cellular responses to various cues — including exposure to radiation — there is a compelling need to develop methods for *in vivo* evaluation of biomarkers and their biochemical transformations during the chain of events leading to final products. Progress in MR sensitivity using hyperpolarization^10^, ^11^ affords the detection of transient biomarkers during their buildup and depletion in enzymatic cascades. These intermediate biomarkers, when detected *in vivo*, can be decisive for evaluating radiation efficiency on an individual basis. A better understanding of the processes involved in radiation‐induced damage signaling, upstream of the endpoints, will allow an improved monitoring of the outcome of radiotherapy based on sequences of enzyme‐controlled transformations.

This radiation‐monitoring approach may allow, in the case of high dose‐rate radiation, for fine tuning of the doses and of the sequences of radiation pulses for personalized therapy, as well as for the development of cotherapies to increase treatment efficacy and to limit radiotherapy side effects.^12^

Molecular imaging techniques are used to monitor functional and metabolic biomarkers for better orienting and monitoring personalized radiotherapeutic strategies.^13^ Reliable biomarkers that mirror the effects of radiotherapy in terms of efficacy, side effects, and underlying mechanisms are a prerequisite. Some can be identified by “systems medicine” tools^14^ for minimally invasive screening in blood. New perspectives for accurate investigation of cancer‐related biomarkers are now provided by liquid biopsy, which allows isolation of circulating tumor cells, tumor DNA, and exosomes, as a rich and more specific source of genomic and proteomic information for cancer patients.^15^ The technological advance brought by next‐generation sequencing (NGS) for DNA and, more recently, RNA, supports high‐throughput evaluation of single‐nucleotide polymorphisms (SNPs) and accurate prognosis of radiotherapy outcome.^16^, ^17^

UV‐Vis or fluorescent spectroscopic methods for the detection of biomarkers^18^ rely on the availability of molecules featuring chromophores or the introduction of biocompatible molecules bearing chromophores. Molecular imaging using Positron Emission Tomography (PET) and Magnetic Resonance (MR) have the potential to orient radiotherapy by observing its effects *in vivo*. In terms of the minimum quantity of a tracer molecule needed for detection, MR‐based techniques using stable isotopes are less sensitive than PET. However, they are more flexible in terms of setup and can be used to image therapy effects noninvasively as often as clinically necessary after initial and subsequent radiotherapy sessions.^19^ Chemotherapy, photodynamic therapy, or other treatments can be equally followed.^11^, ^20^, ^21^, ^22^ Molecular monitoring of radiation effects by MR allows for translation of laboratory research to the clinic, as parallel in‐cell*, ex vivo* (on biological samples stemming from patients) and *in vivo* imaging of the effects of radiation can be conducted with the same approach.

Biomarkers can be used for improving image‐guided interventions. This raises the need to identify endogenous molecules that provide information on the effects induced by ionizing radiation in due time, that is, before toxic effects arise, help predict individualized radiation sensitivity and treatment outcome.^23^ Various strategies have been developed to improve the outcome of radiotherapy by increasing efficiency at the tumor site and decreasing overall toxicity, for example, defining shorter time intervals between radiation exposures in order to apply the required total therapeutic dose quickly, without allowing exposed cells to recover. Such approaches will benefit from close monitoring via biomarkers. The radiation dose rate is a key radiotherapy parameter that will afford significant improvements of treatment outcome, as physics opens the access to higher radiation intensities and shorter timescales.

## 2 High dose‐rate radiation sources

In recent years, short‐pulse high‐power lasers^24^ have shown their ability to drive particle beams for cells and tissue radiation. The results obtained so far in studying their biological effects are encouraging: laser‐accelerated particle beams have the potential of finding use in radiotherapy.^25^ Laser‐driven ionizing radiation (gamma beams, x rays, ions, or electrons) can be generated in pulses with durations of fs to ns, depending on the time length of the driving laser pulse, with doses up to several Gy/pulse. A petawatt‐class laser beam delivers ca. 10^20^ photons in tens of femtoseconds to a solid target, which can, in turn, generate 10^10^–10^11^ ions with energies higher than 10 MeV/nucleon, depending on the laser acceleration mechanism.^26^

Radiation can also be delivered on short timescales (e.g., milliseconds) using classical accelerators. Such pulses have been demonstrated using a superconducting nanoscope in conjunction with TANDEM accelerators for proton irradiation.^27^, ^28^ Currently, development efforts are aimed at reducing the size and the costs of particle accelerators.^29^ It is too early to predict whether the very short distance acceleration specific to high‐power laser‐based particle accelerators^30^ will be conveyed in advantages in terms of compactness.

As laser‐driven ions penetrate cells, they produce free radicals, mainly via water radiolysis.^5^, ^31^, ^32^ A dose of 1 Gy delivered in one shot equates to a dose rate of 10^9^ Gy/s. High energies can be attained with the current state of the art for laser‐driven proton acceleration (Table 1): protons were accelerated to 93 MeV at GIST in South Korea^33^ and nearly 100 MeV at RAL in the United Kingdom.^34^ The obtained high energies are auspicious for the application of laser‐based ion acceleration in radiotherapy.

**Table 1:** Radiobiology experiments using short‐pulse lasers and classically generated proton beams ordered by the delivered dose rate.

	Source	Proton energy	Dose rate	Proton pulse duration	Reference
High Power Laser System	J‐Karen (17 TW, 35 fs)	2.5 MeV	0.01 Gy/ns	15 ns	Yogo et al^38^
	Draco (60 TW, 30 fs) Draco (100 TW, 30 fs)	15 MeV 20 MeV	0.01 Gy/ns	2 ns	Zeil et al^39^ Kraft et al^40^
	Arcturus (200 TW, 30 fs)	2.1 MeV	0.03 Gy/ns	1 ns	Raschke et al^36^
	Taranis (30 TW, 700 fs)	4.5 MeV	1 Gy/ns	1 ns	Doria et al^41^
	Atlas (30 TW, 30 fs)	5.2 MeV	4.6 Gy/ns	1 ns	Bin et al^42^
	PICO2000 (100 TW, 1.3 ps)	10 MeV	1 Gy/ns	1 ns	Manti et al^43^
Classical Accelerator	MGH Francis H. Burr Proton Beam Therapy Center, USA	230 MeV	2 Gy/min	200 ms	Schlegel et al^44^

On the side of experimental evidence using radiation from classical accelerators, recent research conducted *in vivo* on animal models concluded that the use of intermediate‐to‐high radiation dose rates (“FLASH”) on the order of 1 Gy/10 ms, obtained with conventional particle accelerators is a good radiation strategy to spare healthy tissue while remaining effective on tumor tissue.^35^, ^36^ Such effects are found at the level of radiation doses used in clinical practice.^37^ Laser sources able to deliver proton pulses corresponding to the radiation levels of Gy on timescales of ns offer a new tool for the study of radiobiological effects as a function of the dose‐rates.

The relationship between the persistence of the free radicals generated on ns time scales or even faster and the inflicted cell damage is to be explored, as bursts of free radicals on ns timescales were difficult to induce until recently. The molecular effects of radiation pulses delivered to cells on these timescales are anticipated to offer new therapeutic promises.^38^

Decreases in tumor radiosensitivity by transient oxygen depletion following a short‐pulse irradiation^39^ are an issue to be considered in high dose‐rate radiation experiments. The time to reverse oxygen depletion within tumors is estimated to be> 20 s at distances of about 100 µm from a tumor blood vessel. In pulsed radiotherapy, the timing is best planned to deliver the pulses once the oxygen concentration recovers in the tumor. The abundance of oxygen allows radiation‐induced free radicals (e.g., hydroxyl) to react with oxygen and form new radicals (e.g., peroxyl) that cause permanent damage to DNA, hence rendering radiotherapy more effective. The free radical‐mediated differences between cellular behavior under radiotherapy in oxygen‐rich and oxygen‐starvation conditions require that short‐pulse laser‐based radiobiology experiments be carried out in both types of environments.

### 2.A. Short pulses and high dose‐rate proton beams

Several relevant experiments of cell radiation by pulsed beams using laser‐driven and conventionally accelerated protons are presented in Table 1. The average energy and particle pulse duration are pertinent for the penetration depth and the dose rate of radiation, respectively.

Laser sources are expected, in the near future, to deliver proton pulses corresponding to radiation levels of Gy on timescales of hundreds of ps. High‐density protons delivered in clusters are thought to manifest increased linear energy transfer (LET) compared to isolated protons. Via computer simulations, the convergence of proton tracks was anticipated to have damaging effects similar to those of high LET radiation.^40^ The impact of clustered protons on cell survival and DNA integrity^41^, ^42^ has been investigated experimentally, yet molecular effects are still to be assessed in this type of experiments.

Emerging studies comparing the effects of high dose‐rate laser‐driven radiation and low dose‐rate radiation showed that imaging techniques have to be further developed to address cellular responses to ionizing radiation exposure in order to help decide on the most effective strategy for applying radiation pulses.^43^, ^44^

### 2.B. Beyond laser‐generated protons: heavy ions and other types of radiation

Investigations of the radiobiological effects of ions heavier than protons have been carried out since decades using conventional accelerators. It is well known that heavy ions based radiotherapy produces stronger biological effects than proton‐beam therapy. So far, radiobiology research has been carried out using low dose‐rate heavy ions radiation, but the biological effects of high dose‐rate heavy ions radiation are unknown due to the complete lack of experiments. It is noteworthy that experiments with laser‐accelerated carbon ions are ongoing in petawatt‐class facilities. Medical applications require ions with energies up to a few hundred MeV per nucleon, whereas, at the moment, laser‐accelerated ions, such as carbon‐12, can reach only tens of MeV per nucleon. About 10 years ago, researchers were able to generate carbon ions with maximum energy of 6 MeV/n using a 30 TW‐class laser at the Max Born Institute in Germany.^45^ Recently, at the Central Laser Facility of Rutherford Appleton Laboratory in the United Kingdom, carbon ions with energy up to 25 MeV/n were obtained by employing the 400 TW GEMINI laser focused to an intensity of about 5 × 10^20^ W/cm^2^ on a target consisting of an ultrathin carbon foil.^46^ Such ion energies can already serve for radiobiology experiments *in vitro* to study the effect of ultra‐high dose‐rates (~10^9^ Gy/s) prior to medical applications. Most likely, with the next generation of 10 PW‐class lasers, for example, at ELI‐NP,^47^, ^48^ the carbon ion energy is expected to reach hundreds of MeV/n, as computer simulations predict.^49^, ^50^ There is a significant interest in radiotherapeutic application of electrons accelerated by high power lasers in gaseous targets. Laser‐plasma accelerators produce femtosecond electron bunches^51^ that are quasi mono‐energetic with energies in the range of tens of MeV up to a few GeV, and with a pulsed dose‐rate exceeding 10^13^ Gy/s. Results have been recently reported on biological material irradiated with laser generated electrons both *in vitro* 52, 53 and *in vivo.* 54, 55

In order to destroy tumor cells that adapt to radiation, synergy between the effects of subsequent pulses or different types of radiation is taken into consideration in complex radiotherapy‐oriented studies. For the moment, evidence of such synergy was found^56^ using sequential exposures of cells to radiation with low‐ and high‐LET, using radiation beams obtained with classical accelerators at much lower dose‐rates than those provided by high power lasers. Therefore, synergistic radiation effects^57^ on cells exposed to different beams delivered at high dose‐rates are still to be investigated with laser‐accelerated particles and photons.

## 3 Molecular imaging: early detection for high dose‐rate effects

**Figure 1:**
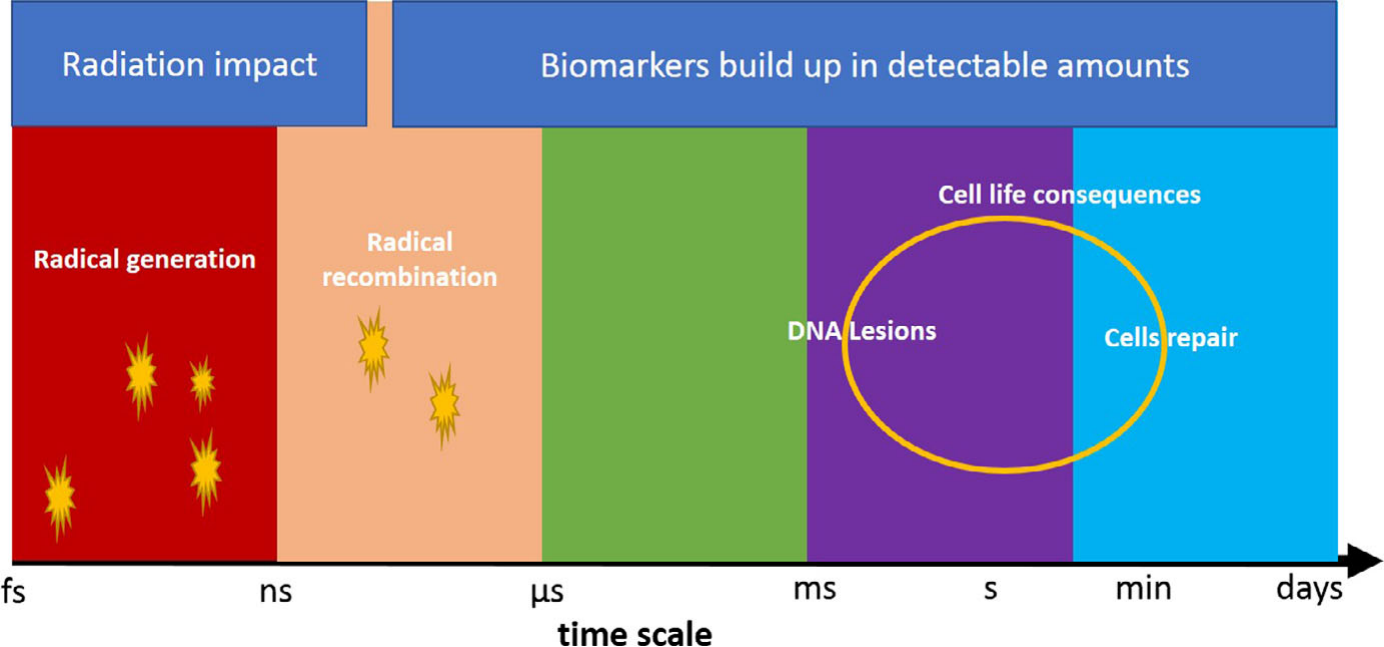
Formation of free radicals in large amounts by the initial impact of radiation defines the timescale beyond which the cell radiation response is triggered. The response can be sensed detecting molecular biomarkers related to the radiation‐induced damage.

Early detection of radiation effects is needed to orient pulsed radiotherapy in real time. The formation of free radicals and reactive molecules either directly by radiolysis or as a cellular response to radiation, if promptly detected, can help in this regard. Due to the timescale on which free radicals are formed and recombine in laser‐stemming radiation (Fig. 1), their detection by noninvasive methods is challenging in cell cultures and even more so in complex organisms. The persisting free radicals generated by high dose‐rate radiation can be measured in cells by: (a) electron spin resonance (ESR) combined with the use of radical‐specific spin traps, provided the latter are biocompatible or (b) spectroscopic methods whereby the effects of the irradiation of free radicals on biomolecules are amenable to *in vivo* monitoring.^19^, ^20^, ^58^

Clinical studies have addressed the use of MRI in the radiotherapy of static organs, for example, for the treatment of brain and prostate cancers.^59^ Structural changes are readily detected by MR imaging based on abundant water and on contrast agents — also based on the uptake of these agents to the tumor site, as in dynamic contrast‐enhanced MRI,^60^ but these reductions in tumor size only occur a few weeks after treatment. MR‐based *in vivo* monitoring of cancer response to radiation is especially sensitive to oxygen presence via mechanisms forming reactive species.^61^ The use of MRI to detect the responses of endogenous molecules present in low concentrations is, however, limited by its low signal‐to‐noise ratio.

Emerging MRI‐guided proton beam therapy (MRgPT) elicits increasing interest, triggered by the already‐implemented real‐time MRI‐guided x‐ray beam therapy (MRXT), as MRI guidance for proton beam therapy will offer an improvement over the conventional proton therapy in terms of toxicity outcomes. The use of MRI‐Cobalt (ViewRay™) for imaging‐oriented radiotherapy was shown to extend cancer survival by up to a factor 2.^62^ MRI‐LINAC prototypes are available for *in vivo* imaging of radiation effects.^63^ Thus, it was shown that there is a significant interest and potential for clinical use of an integrated MRI‐LINAC system.

We comment herein on the potential of using noninvasive magnetic resonance methods to orient pulsed radiation therapy, when the detected concentrations of endogenous molecules are sensitive, directly or indirectly, to the levels of free radicals induced by radiation.^11^, ^58^, ^64^ Imaging at the organism level by MRI can be based on particular biomarkers identified in cells exposed to radiation. The developing MRI‐LINAC and MRgPT systems are expected to profit from the use of biomarker‐based detection.

### 3.A. Hyperpolarized magnetic resonance for the detection of radiation effects

The sensitivity of MR has been recently improved by a factor 10^4^ using “hyperpolarization.”^10^ The most versatile form of hyperpolarized MR uses signal transfer from free radicals to magnetic nuclei by irradiation at electron paramagnetic resonance (EPR) frequencies, an experimental development known as hyperpolarization or Dynamic Nuclear Polarization (DNP). The sensitivity increase derived from free radicals is directed toward the observation of signals of endogenous molecules that would be difficult to detect lacking hyperpolarization, such as the carbon‐13 signal of pyruvate. Considerations related to signal lifetimes or resolution dictate the choice of stable isotopes (protons, carbon‐13, nitrogen‐15, etc.) and molecular groups to be detected.^65^, ^66^ When hyperpolarization can be performed for protons, whose detection is intrinsically more sensitive than that of other spins such as carbon‐13, the gain in signal affords improving the resolution of obtained images. Recent developments show that hyperpolarization can also be transferred from water to the detected biomarkers.^67^, ^68^, ^69^ The observation timescales of the method can be adapted for the durations needed for diagnostic^70^ by carefully selecting the molecular regions for storing magnetization. Parallel studies *in‐cell* 71 and *in vivo* can be carried out using pyruvate or other biomarkers. The DNP‐MR imaging approach has been adapted for the clinic.^72^ Early response to ionizing radiation, which matches the requirements for the short‐pulse laser‐based radiotherapy mentioned above, has been confirmed by MRI in studies conducted *in vivo* within hours after radiation therapy.^73^

**Figure 2:**
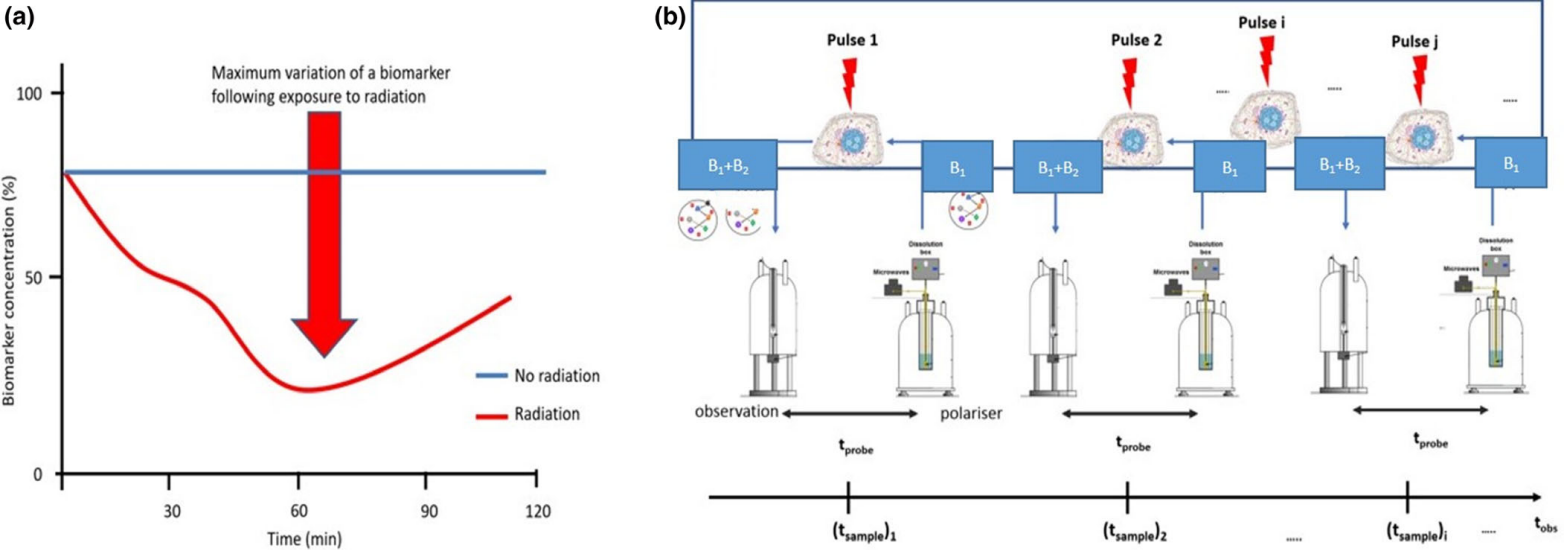
a) Biomarker detection strategy using molecular imaging based on hyperpolarized (DNP‐enhanced) magnetic resonance for following radiotherapy effects. Free radicals and reactive oxygen and nitrogen species produced by radiation‐triggered mechanisms on a timescale of several hours alter the concentration of detected biomarkers. The figure is adapted from Ref. [58] b) Experimental setup for the observation of radiation effects *in cell* serving as a test laboratory for imaging‐oriented radiotherapy *in vivo*. B_1_ and B_2_ are biomolecular markers sensitive to radiation effects exerted via reactive molecular species (free radicals), *t*_probe_ < 5 min for most endogenous molecules, and *t*_obs_ is on the order of tens of minutes to hours. For *in vivo* detection, an MRI scanner replaces the observation spectrometer. DNP, Dynamic Nuclear Polarization.

A strategy for orienting radiation therapy based on hyperpolarized MRI biomarkers has been described in the literature,^58^ as shown in Fig. 2. The strategy is suitable for the optimization of sequences of short‐pulse laser‐generated particles for radiotherapy, in which case the optimal placement of pulsed radiation (red arrow) is to be inferred *in vivo.*

The advantages of this approach have been outlined by a recent work^74^ that substantiated both the solid tumor response and the effects of radiotherapy on healthy tissue in order to survey potential radiation toxicity for areas surrounding the targeted tumor. The response of different types of cancer to therapy has also been monitored using hyperpolarized magnetic resonance, notably in glioma^75^ and squamous cell carcinoma.^76^ This latter type of cancer is one of the first likely targets for radiotherapy based on short‐pulse lasers with particles of low energy. In addition to efficiency at the tumor site, the effects of ionizing radiation on healthy tissue can also be imaged by dynamic nuclear polarization‐enhanced magnetic resonance^77^ and the response quantified in terms of individual metabolites.^78^ Thereby, the follow‐up of radiotherapy by MR can be used to prove the reduced toxicity of ultra‐high dose‐rate radiation.

### 3.B. Nanoparticles and magnetic resonance imaging in the context of radiation therapy

In connection with MRI, nanoparticles are tested for biomarker development and treatment delivery. On the one hand, MRI detection of biomarkers can be improved using nanoparticles. Their large specific surface and the fact that this surface can be functionalized according to the intended use make them ideal candidates for developing biomarker platforms.^79^ The main challenge is to functionalize nanoparticle surfaces to selectively bind a subset of biomarkers. For instance, sensitive detection of a specific DNA sequence was developed using magnetic nanoparticles (MNPs). A significant detection amplification was obtained from multiple binding sites on the MNPs, achieving thousands of DNA molecules on one MNP surface.^80^ Additionally, nanoparticles that can link to biomarkers or accumulate at a certain location within tissues will enhance the image by improving resolution or sensitivity inside tissues. A large number of nanoparticles have been studied in this context. Specifically, Gd‐loaded targeted nanoparticles are used as MRI contrast agents for molecular imaging to detect molecular signatures in various pathologies.^81^

On the other hand, nanoparticles can also deliver targeted chemotherapeutic drugs and enhance MRI imaging of their effects. Thus, nanostructures are expected to be used as targeted theranostic nanoplatforms for both therapeutic and imaging purposes. This is an important aspect in personalized cancer medicine, given their therapeutic potential and their simultaneous ability to track a patient's response to chemotherapy at the same time.^82^ As an example, complex iron oxide nanostructures can be made of three components: a superparamagnetic iron core, a biocompatible polymer coating, and functional moieties. These decorated nanoparticles can function as contrast agents for magnetic resonance imaging due to the iron core and as carrier vehicles for drugs attached to their polymer coating.^83^

Nanoparticles are used to enhance the therapeutic effects of radiation in cancer treatment. Satterlee et al.^84^ reported the use of a special type of nanoparticles (lipid‐calcium phosphate nanoparticles) and ^177^Lu for radiation therapy. The beta decay of ^177^Lu significantly decreased the growth of cancerous cells, as an enhanced permeability and retention (EPR) effect at the tumor site was achieved. Gold nanoparticles can be functionalized with epidermal growth factors (EFG) and labeled with ^111^In.^85^ Gold nanoparticles demonstrated radio‐sensitizing potential and improved localization of the radiation dose within the tumor.^86^ Moreover, gold nanoparticles can interact with x rays or ion beams producing electrons which can further modulate the radiation treatment.^87^

## 4 Summary

High‐power lasers are unique sources for radiation featuring new interactions with living matter and very promising opportunities for radiotherapy. The effects of high dose‐rate radiation on cells and organisms need to be characterized using endogenous biomarkers in order to be fully exploited for spatially and time‐controlled radiotherapy. Using DNP to enhance the sensitivity of MRI, radiation‐induced perturbations mediated by free radicals can be followed inside cells. The irradiation of free radicals with microwave frequency can also be used *in vivo* 88 to generate DNP‐MRI images. The differences between high dose‐rate and classical radiation, with the former being increasingly regarded as less toxic to healthy tissue, can be monitored by hyperpolarized MRI in real time, on the timescale of biological reactions. There are important advantages to guiding laser‐based radiation with DNP‐MRI: (a) sequences of radiation pulses guided by DNP‐MRI can be optimized *in cells* and subsequently used *in vivo* based on this optimization; (b) the difference between high dose‐rate radiation and classically generated radiation resides in the formation of reactive molecular species whose dynamic transformations can be followed by DNP‐MRI; (c) short‐pulse radiation effects are detected in a timely manner. *In vivo*, functional effects can be sensed by biomarkers hours after treatment, well before any observable structural morphologic changes would occur in tumors — that is, anticipating tumor shrinkage. With early monitoring of its effectiveness and toxicity at hand, radiotherapy may become, in numerous cases, the first clinical approach to be considered.

The pulsed nature of the laser‐generated radiation, which exerts its effect in short bursts, may redefine the way of anticipating the biological effectiveness and the toxicity of different types of particle beams. Notwithstanding the fact that the same quantity of energy is delivered per unit mass to living tissues or cells by the same particle type, the effectiveness of the dose can depend on the pulse duration, as free radicals in water are known to recombine on fast timescales. It emerges that, with the development of ultrashort radiation pulses, the inclusion of a time dimension in the description of impinging radiation is needed to anticipate the biological effect of radiation doses. Consequently, the picture describing the classical mechanisms by means of which ionizing radiation exerts biological effects can be revisited considering the ultra‐short period of radiation action, in the case of high‐power laser‐based radiation. This can have beneficial consequences for exploiting the observed mechanistic effects in radiotherapy, in terms of deleterious effects for tumors and sparing of the healthy tissue.

## Conflict of interest

The authors have no conflicts to disclose.

## References

[1] Delaney G , Jacob S , Featherstone C , Barton M . The role of radiotherapy in cancer treatment. Cancer. 2005;104:1129–1137. 16080176 10.1002/cncr.21324

[2] Barton MB , Frommer M , Shafiq J . Role of radiotherapy in cancer control in low‐income and middle‐income countries. Lancet Oncol. 2006;7:584–595. 16814210 10.1016/S1470-2045(06)70759-8

[3] Tyldesley S , Delaney G , Foroudi F , Barbera L , Kerba M , Mackillop W . Estimating the need for radiotherapy for patients with prostate, breast, and lung cancers: verification of model estimates of need with radiotherapy utilization data from British Columbia. Int J Radiat Oncol*Biol*Phy. 2011;79:1507–1515. 10.1016/j.ijrobp.2009.12.070 20605338

[4] Gelband H , Jha P , Sankaranarayanan R , Horton S . eds. Cancer: Disease Control Priorities, Third Edition (Volume 3). Washington (DC): The International Bank for Reconstruction and Development / The World Bank; 2015. http://www.ncbi.nlm.nih.gov/books/NBK343628/. Accessed March 28, 2018. 26913318

[5] Giulietti A . Laser‐Driven Particle Acceleration Towards Radiobiology and Medicine. Cham: Springer; 2016.

[6] Schieber M , Chandel NS . ROS function in redox signaling and oxidative stress. Curr Biol. 2014;24:R453–462. 24845678 10.1016/j.cub.2014.03.034 PMC4055301

[7] Finkel T . From sulfenylation to sulfhydration: what a thiolate needs to tolerate. Sci Signal. 2012;5:pe10–pe10. 22416275 10.1126/scisignal.2002943

[8] Manda G , Nechifor MT , Neagu T‐M . Reactive oxygen species, cancer and anti‐cancer therapies. Curr Chem Biol. 2009;3:342–366.

[9] Cuadrado A , Manda G , Hassan A , et al. Transcription factor NRF2 as a therapeutic target for chronic diseases: a systems medicine approach. Pharmacol Rev. 2018;70:348–383. 29507103 10.1124/pr.117.014753

[10] Ardenkjaer‐Larsen JH , Fridlund B , Gram A , et al. Increase in signal‐to‐noise ratio of > 10,000 times in liquid‐state NMR. Proc Natl Acad Sci USA. 2003;100:10158–10163. 12930897 10.1073/pnas.1733835100 PMC193532

[11] Kurhanewicz J , Vigneron DB , Ardenkjaer‐Larsen JH , et al. Hyperpolarized 13C MRI: path to clinical translation in oncology. Neoplasia. 2018;21:1–16. 30472500 10.1016/j.neo.2018.09.006 PMC6260457

[12] Thomas M , Sukhai MA , Zhang T , et al. Integration of technical, bioinformatic, and variant assessment approaches in the validation of a targeted next‐generation sequencing panel for myeloid malignancies. Arch Pathol Lab Med. 2017;141:759–775. 28557600 10.5858/arpa.2016-0547-RA

[13] Chen HHW , Kuo MT . Improving radiotherapy in cancer treatment: promises and challenges. Oncotarget. 2017;8:62742–62758. 28977985 10.18632/oncotarget.18409 PMC5617545

[14] Gonzalez‐Angulo AM , Hennessy BTJ , Mills GB . Future of personalized medicine in oncology: a systems biology approach. J Clin Oncol. 2010;28:2777–2783. 20406928 10.1200/JCO.2009.27.0777 PMC2881854

[15] Palmirotta R , Lovero D , Cafforio P , et al. Liquid biopsy of cancer: a multimodal diagnostic tool in clinical oncology. Ther Adv Med Oncol. 2018;10:1758835918794630. 30181785 10.1177/1758835918794630 PMC6116068

[16] Tinhofer I , Niehr F , Konschak R , et al. Next‐generation sequencing: hype and hope for development of personalized radiation therapy? Radiat Oncol. 2015;10:183. 26316159 10.1186/s13014-015-0481-x PMC4554356

[17] Bibault J‐E , Tinhofer I . The role of next‐generation sequencing in tumoral radiosensitivity prediction. Clin Transl Radiat Oncol. 2017;3:16–20. 29658008 10.1016/j.ctro.2017.03.002 PMC5893518

[18] Cheng Z , Yan X , Sun X , Shen B , Gambhir SS . Tumor molecular imaging with nanoparticles. Engineering. 2016;2:132–140.

[19] Jones KM , Michel KA , Bankson JA , Fuller CD , Klopp AH , Venkatesan AM . Emerging magnetic resonance imaging technologies for radiation therapy planning and response assessment. Int J Radiat Oncol Biol Phys. 2018;101:1046–1056. 30012524 10.1016/j.ijrobp.2018.03.028 PMC9671538

[20] Bankson JA , Walker CM , Ramirez MS , et al. Kinetic modeling and constrained reconstruction of hyperpolarized [1‐13C]‐Pyruvate offers improved metabolic imaging of tumors. Cancer Res. 2015;75:4708–4717. 26420214 10.1158/0008-5472.CAN-15-0171 PMC4651725

[21] Yu W , Chen Y , Dubrulle J , et al. Cisplatin generates oxidative stress which is accompanied by rapid shifts in central carbon metabolism. Sci Rep. 2018;8:4306. 29523854 10.1038/s41598-018-22640-y PMC5844883

[22] Manda G , Hinescu ME , Neagoe IV , et al. Emerging therapeutic targets in oncologic photodynamic therapy. Curr Pharm Des. 2018;24:5268–5295. 30674246 10.2174/1381612825666190122163832

[23] Shukla HD . Novel genomics and proteomics based biomarkers to predict radiation response and normal radiotoxicity in cancer patients for personalized medicine. J Cancer Clin Trials. 2016;1:1–3.

[24] Strickland D , Mourou G . Compression of amplified chirped optical pulses. Opt Commun. 1985;55:447–449.

[25] Hofmann KM , Schell S , Wilkens JJ . Laser‐driven beam lines for delivering intensity modulated radiation therapy with particle beams. J Biophotonics. 2012;5:903–911. 22930653 10.1002/jbio.201200078 PMC3672655

[26] Tajima T , Habs D , Yan X . Laser acceleration of Ions for radiation therapy. Rev Accl Sci Tech. 2009;02:201–228.

[27] Zlobinskaya O , Siebenwirth C , Greubel C , et al. The effects of ultra‐high dose rate proton irradiation on growth delay in the treatment of human tumor xenografts in nude mice. Radiat Res. 2014;181:177–183. 24524347 10.1667/RR13464.1

[28] Schmid TE , Dollinger G , Hable V , et al. Relative biological effectiveness of pulsed and continuous 20 MeV protons for micronucleus induction in 3D human reconstructed skin tissue. Radiother Oncol. 2010;95:66–72. 20347168 10.1016/j.radonc.2010.03.010

[29] Margarone D , Cirrone GAP , Cuttone G , et al. ELIMAIA: a laser‐driven ion accelerator for multidisciplinary applications. Quantum Beam Sci. 2018;2:8.

[30] Giulietti A , Tajima T .Lasers Offer New Tools to Radiobiology and Radiotherapy. In:2016 :1–15.

[31] Desouky O , Ding N , Zhou G . Targeted and non‐targeted effects of ionizing radiation. J Radiat Res Appl Sci. 2015;8:247–254.

[32] Reisz JA , Bansal N , Qian J , Zhao W , Furdui CM . Effects of ionizing radiation on biological molecules—mechanisms of damage and emerging methods of detection. Antioxid Redox Signal. 2014;21:260–292. 24382094 10.1089/ars.2013.5489 PMC4060780

[33] Kim IJ , Pae KH , Choi IW , et al. Radiation pressure acceleration of protons to 93 MeV with circularly polarized petawatt laser pulses. Phys Plasmas. 2016;23:070701.

[34] Higginson A , Gray RJ , King M , et al. Near‐100 MeV protons via a laser‐driven transparency‐enhanced hybrid acceleration scheme. Nat Commun. 2018;9:724. 29463872 10.1038/s41467-018-03063-9 PMC5820283

[35] Favaudon V , Caplier L , Monceau V , et al. Ultrahigh dose‐rate FLASH irradiation increases the differential response between normal and tumor tissue in mice. Sci Transl Med. 2014;6:245ra93. 10.1126/scitranslmed.3008973 25031268

[36] Vozenin M‐C , De Fornel P , Petersson K , et al. The advantage of FLASH radiotherapy confirmed in Mini‐pig and Cat‐cancer patients. Clin Cancer Res. 2019;25:35–42. 29875213 10.1158/1078-0432.CCR-17-3375

[37] Montay‐Gruel P , Petersson K , Jaccard M , et al. Irradiation in a flash: unique sparing of memory in mice after whole brain irradiation with dose rates above 100Gy/s. Radiother Oncol. 2017;124:365–369. 28545957 10.1016/j.radonc.2017.05.003

[38] Raschke S , Spickermann S , Toncian T , et al. Ultra‐short laser‐accelerated proton pulses have similar DNA‐damaging effectiveness but produce less immediate nitroxidative stress than conventional proton beams. Sci Rep. 2016;6:32441. 27578260 10.1038/srep32441 PMC5006042

[39] Wilson P , Jones B , Yokoi T , Hill M , Vojnovic B . Revisiting the ultra‐high dose rate effect: implications for charged particle radiotherapy using protons and light ions. Br J Radiol. 2012;85:e933–e939. 22496068 10.1259/bjr/17827549 PMC3474025

[40] Fourkal E , Velchev I , Ma C‐M , Fan J . Linear energy transfer of proton clusters. Phys Med Biol. 2011;56:3123–3136. 21521908 10.1088/0031-9155/56/10/015

[41] Schmid TE , Dollinger G , Hauptner A , et al. No evidence for a different RBE between pulsed and continuous 20 MeV protons. Radiat Res. 2009;172:567–574. 19883224 10.1667/RR1539.1

[42] Schmid TE , Greubel C , Hable V , et al. Low LET protons focused to submicrometer shows enhanced radiobiological effectiveness. Phys Med Biol. 2012;57:5889–5907. 22955045 10.1088/0031-9155/57/19/5889

[43] Minafra L , Bravata V , Cammarata FP , Forte GI . Radiation therapy towards laser‐driven particle beams: an “OMICS” approach in radiobiology In: Giulietti A, ed. Laser‐Driven Particle Acceleration Towards Radiobiology and Medicine. New York: Springer; 2016:67–98.

[44] Yogo A , Sato K , Nishikino M , et al. Measurement of DNA double‐strand break yield in human cancer cells by high‐current, short‐duration bunches of laser‐accelerated protons. Jpn J Appl Phys. 2011;50:106401.

[45] Henig A , Steinke S , Schnürer M , et al. Radiation‐pressure acceleration of ion beams driven by circularly polarized laser pulses. Phys Rev Lett. 2009;103:245003. 20366205 10.1103/PhysRevLett.103.245003

[46] Scullion C , Doria D , Romagnani L , et al. Polarization dependence of bulk Ion acceleration from ultrathin foils irradiated by high‐intensity ultrashort laser pulses. Phys Rev Lett. 2017;119:054801. 28949740 10.1103/PhysRevLett.119.054801

[47] Negoita F , Roth M , Thirolf PG , et al. Laser driven nuclear physics at Eli‐Np. Rom Rep Phys. 2016;68:S37–S144.

[48] Asavei T , Tomut M , Bobeica M , et al. Materials in extreme environments for energy, accelerators and space applications at ELI‐NP. Rom Rep Phys. 2016;68:S275–S347.

[49] Esirkepov T , Borghesi M , Bulanov SV , Mourou G , Tajima T . Highly efficient relativistic‐ion generation in the laser‐piston regime. Phys Rev Lett. 2004;92:175003. 15169160 10.1103/PhysRevLett.92.175003

[50] Qiao B , Zepf M , Borghesi M , Geissler M . Stable GeV ion‐beam acceleration from thin foils by circularly polarized laser pulses. Phys Rev Lett. 2009;102:145002. 19392446 10.1103/PhysRevLett.102.145002

[51] Rigaud O , Fortunel NO , Vaigot P , et al. Exploring ultrashort high‐energy electron‐induced damage in human carcinoma cells. Cell Death Dis. 2010;1:e73. 21364677 10.1038/cddis.2010.46 PMC3032345

[52] Laschinsky L , Baumann M , Beyreuther E , et al. Radiobiological effectiveness of laser accelerated electrons in comparison to electron beams from a conventional linear accelerator. J Radiat Res. 2012;53:395–403. 22739009 10.1269/jrr.11080

[53] Andreassi MG , Borghini A , Pulignani S , et al. Radiobiological effectiveness of ultrashort laser‐driven Electron bunches: micronucleus frequency, telomere shortening and cell viability. Radiat Res. 2016;186:245–253. 27439449 10.1667/RR14266.1

[54] Brüchner K , Beyreuther E , Baumann M , Krause M , Oppelt M , Pawelke J . Establishment of a small animal tumour model for in vivo studies with low energy laser accelerated particles. Radiat Oncol. 2014;9:57. 24533586 10.1186/1748-717X-9-57 PMC3936820

[55] Oppelt M , Baumann M , Bergmann R , et al. Comparison study of in vivo dose response to laser‐driven versus conventional electron beam. Radiat Environ Biophys. 2015;54:155–166. 25600561 10.1007/s00411-014-0582-1

[56] Ngo FQ , Blakely EA , Tobias CA , Chang PY , Lommel L . Sequential exposures of mammalian cells to low‐ and high‐LET radiations. II. As a function of cell‐cycle stages. Radiat Res. 1988;115:54–69. 3393635

[57] DeLaney TF , Liebsch NJ , Pedlow FX , et al. Phase II study of high‐dose photon/proton radiotherapy in the management of spine sarcomas. Int J Radiat Oncol Biol Phys. 2009;74:732–739. 19095372 10.1016/j.ijrobp.2008.08.058 PMC2734911

[58] Sandulache VC , Chen Y , Lee J , et al. Evaluation of hyperpolarized [1‐C‐13]‐pyruvate by magnetic resonance to detect ionizing radiation effects in real time. PLoS ONE. 2014;9:e87031. 24475215 10.1371/journal.pone.0087031 PMC3903593

[59] Koivula L , Wee L , Korhonen J . Feasibility of MRI‐only treatment planning for proton therapy in brain and prostate cancers: dose calculation accuracy in substitute CT images. Med Phys. 2016;43:4634. 27487880 10.1118/1.4958677

[60] Sandulache VC , Hobbs BP , Mohamed RAS , et al. Dynamic contrast‐enhanced MRI detects acute radiotherapy‐induced alterations in mandibular microvasculature: prospective assessment of imaging biomarkers of normal tissue injury. Sci Rep. 2016;6:29864. 27499209 10.1038/srep29864 PMC4976364

[61] Schroeder M , Laustsen C . Imaging oxygen metabolism with hyperpolarized magnetic resonance: a novel approach for the examination of cardiac and renal function. Biosci Rep. 2017;37:BSR20160186. 27899435 10.1042/BSR20160186 PMC5270319

[62] Metcalfe P , Liney GP , Holloway L , et al. The potential for an enhanced role for MRI in radiation‐therapy treatment planning. Technol Cancer Res Treat. 2013;12:429–446. 23617289 10.7785/tcrt.2012.500342 PMC4527434

[63] Oborn BM , Dowdell S , Metcalfe PE , Crozier S , Mohan R , Keall PJ . Future of medical physics: real‐time MRI‐guided proton therapy. Med Phys. 2017;44:e77–e90. 28547820 10.1002/mp.12371

[64] Kurhanewicz J , Vigneron DB , Brindle K , et al. Analysis of cancer metabolism by imaging hyperpolarized nuclei: prospects for translation to clinical research. Neoplasia. 2011;13:81–97. 21403835 10.1593/neo.101102 PMC3033588

[65] Cavadini S , Vasos PR . Singlet states open the way to longer time‐scales in the measurement of diffusion by NMR spectroscopy. Concept Magn Reson Part A. 2008;32A:68–78.

[66] Sadet A , Fernandes L , Kateb F , Balzan R , Vasos PR . Long‐lived coherences: improved dispersion in the frequency domain using continuous‐wave and reduced‐power windowed sustaining irradiation. J Chem Phys. 2014;141:054203. 25106580 10.1063/1.4891565

[67] Harris T , Szekely O , Frydman L . On the potential of hyperpolarized water in biomolecular NMR studies. J Phys Chem B. 2014;118:3281–3290. 24417324 10.1021/jp4102916 PMC5040483

[68] Kurzbach D , Canet E , Flamm AG , et al. Investigation of intrinsically disordered proteins through exchange with hyperpolarized water. Angew Chem Int Ed. 2017;56:389–392. 10.1002/anie.201608903 27918140

[69] Nastasa V , Stavarache C , Hanganu A , et al. Hyperpolarised NMR to follow water proton transport through membrane channels via exchange with biomolecules. Faraday Discuss. 2018;209:67–82. 29989626 10.1039/c8fd00021b

[70] Vasos PR , Comment A , Sarkar R , et al. Long‐lived states to sustain hyperpolarized magnetization. Proc Natl Acad Sci U S A. 2009;106:18469–18473. 19841270 10.1073/pnas.0908123106 PMC2774001

[71] Balzan R , Fernandes L , Pidial L , Comment A , Tavitian B , Vasos PR . Pyruvate cellular uptake and enzymatic conversion probed by dissolution DNP‐NMR: the impact of overexpressed membrane transporters. Magn Reson Chem. 2017;55:579–583. 27859555 10.1002/mrc.4553

[72] Nelson SJ , Kurhanewicz J , Vigneron DB , et al. Metabolic imaging of patients with prostate cancer using hyperpolarized [1‐13C]Pyruvate. Sci Transl Med. 2013;5:198ra108–198ra108. 10.1126/scitranslmed.3006070 PMC4201045 23946197

[73] Chen AP , Chu W , Gu Y‐P , Cunnhingham CH . Probing early tumor response to radiation therapy using hyperpolarized [1‐13C]pyruvate in MDA‐MB‐231 xenografts. PLoS ONE. 2013;8:1–13. 10.1371/journal.pone.0056551 PMC3570408 23424666

[74] Lai SY , Fuller CD , Bhattacharya PK , Frank SJ . Metabolic imaging as a biomarker of early radiation response in tumors. Clin Cancer Res. 2015;21:4996–4998. 26232369 10.1158/1078-0432.CCR-15-1214 PMC4644683

[75] Day SE , Kettunen MI , Cherukuri MK , et al. Detecting response of rat C6 glioma tumors to radiotherapy using hyperpolarized [1‐C‐13]Pyruvate and C‐13 magnetic resonance spectroscopic imaging. Magn Reson Med. 2011;65:557–563. 21264939 10.1002/mrm.22698 PMC3690628

[76] Saito K , Matsumoto S , Takakusagi Y , et al. C‐13‐MR spectroscopic imaging with hyperpolarized [1‐C‐13]Pyruvate detects early response to radiotherapy in SCC tumors and HT‐29 Tumors. Clin Cancer Res. 2015;21:5073–5081. 25673698 10.1158/1078-0432.CCR-14-1717 PMC8363083

[77] Thind K , Chen A , Friesen‐Waldner L , et al. Detection of radiation‐induced lung injury using hyperpolarized 13C magnetic resonance spectroscopy and imaging. Magn Reson Med. 2013;70:601–609. 23074042 10.1002/mrm.24525

[78] Thind K , Jensen MD , Hegarty E , et al. Mapping metabolic changes associated with early radiation induced lung injury post conformal radiotherapy using hyperpolarized C‐13‐pyruvate Magnetic resonance spectroscopic imaging. Radiother Oncol. 2014;110:317–322. 24440041 10.1016/j.radonc.2013.11.016

[79] Geho DH , Jones CD , Petricoin EF , Liotta LA . Nanoparticles: potential biomarker harvesters. Curr Opin Chem Biol. 2006;10:56–61. 16413816 10.1016/j.cbpa.2006.01.003

[80] Jin M , Liu X , van den Berg A , Zhou G , Shui L . Ultrasensitive DNA detection based on two‐step quantitative amplification on magnetic nanoparticles. Nanotechnology. 2016;27:335102. 27378514 10.1088/0957-4484/27/33/335102

[81] Huang C‐H , Tsourkas A . Gd‐based macromolecules and nanoparticles as magnetic resonance contrast agents for molecular imaging. Curr Top Med Chem. 2013;13:411–421. 23432004 10.2174/1568026611313040002 PMC3761358

[82] Ma Y‐Y , Jin K‐T , Wang S‐B , et al. Molecular imaging of cancer with nanoparticle‐based theranostic probes. Contrast Media Mol Imaging. 2017;2017:1026270. 29097909 10.1155/2017/1026270 PMC5612740

[83] Yigit MV , Moore A , Medarova Z . Magnetic nanoparticles for cancer diagnosis and therapy. Pharm Res. 2012;29:1180–1188. 22274558 10.1007/s11095-012-0679-7 PMC3734862

[84] Satterlee AB , Rojas JD , Dayton PA , Huang L . Enhancing nanoparticle accumulation and retention in desmoplastic tumors via vascular disruption for internal radiation therapy. Theranostics. 2017;7:253–269. 28042332 10.7150/thno.16681 PMC5197062

[85] Song L , Falzone N , Vallis KA . EGF‐coated gold nanoparticles provide an efficient nano‐scale delivery system for the molecular radiotherapy of EGFR‐positive cancer. Int J Radiat Biol. 2016;92:716–723. 26999580 10.3109/09553002.2016.1145360 PMC5116916

[86] Kwatra D , Venugopal A , Anant S . Nanoparticles in radiation therapy: a summary of various approaches to enhance radiosensitization in cancer. *Translational* . Can Res. 2013;2:330–342‐342.

[87] Haume K , Rosa S , Grellet S , et al. Gold nanoparticles for cancer radiotherapy: a review. Cancer Nanotechnol. 2016;7:8. 27867425 10.1186/s12645-016-0021-x PMC5095165

[88] Ito S , Hyodo F . Dynamic nuclear polarization‐magnetic resonance imaging at low ESR irradiation frequency for ascorbyl free radicals. Sci Rep. 2016;6:21407. 26892591 10.1038/srep21407 PMC4759784

